# Chronic fatigue in childhood cancer survivors is associated with lifestyle and psychosocial factors; a DCCSS LATER study

**DOI:** 10.1016/j.esmoop.2023.102044

**Published:** 2023-11-02

**Authors:** A. Penson, I. Walraven, E. Bronkhorst, M.A. Grootenhuis, H. Maurice-Stam, I. de Beijer, M. van der Heiden-van der Loo, W.J.E. Tissing, H.J.H. van der Pal, A.C.H. de Vries, D. Bresters, C.M. Ronckers, M.M. van den Heuvel-Eibrink, S. Neggers, B.A.B. Versluys, M. Louwerens, S.M.F. Pluijm, N. Blijlevens, E. van Dulmen-den Broeder, L.C.M. Kremer, H. Knoop, J. Loonen, Dutch LATER study group

**Affiliations:** 1 Radboud University Medical Center, Center of Expertise for Cancer Survivorship, Department of Hematology, Nijmegen; 2 Department for Health Evidence, Radboud University Medical Center, Nijmegen; 3 Princess Máxima Center for Pediatric Oncology, Utrecht; 4 Department of Pediatric Oncology/Hematology, Beatrix Children’s Hospital/University of Groningen/University Medical Center Groningen, Groningen; 5 Department of Pediatric Oncology, Erasmus Medical Center, Rotterdam, The Netherlands; 6 Division of Childhood Cancer Epidemiology, Institute of Medical Biostatistics, Epidemiology and Informatics (IMBEI), University Medical Center of the Johannes Gutenberg University, Mainz, Germany; 7 Wilhelmina Children’s Hospital, UMCU, Utrecht; 8 Department of Medicine, Section Endocrinology, Erasmus Medical Center, Rotterdam; 9 Leiden University Medical Center, Department of Internal Medicine, Leiden; 10 Department of Pediatric Oncology/Hematology, Amsterdam University Medical Center, Amsterdam; 11 Department of Pediatric Oncology, Emma Children’s Hospital, University of Amsterdam, Amsterdam; 12 Department of Medical Psychology, Amsterdam University Medical Centers, University of Amsterdam, Amsterdam Public Health Research Institute, Amsterdam, The Netherlands

**Keywords:** childhood cancer, survivorship, chronic fatigue, late effects

## Abstract

**Background:**

The purpose of this study was to determine factors associated with chronic fatigue (CF) in childhood cancer survivors (CCS).

**Patients and methods:**

Participants were included from the Dutch Childhood Cancer Survivor Study (DCCSS) LATER cohort, a nationwide cohort of CCS (≥5 years after diagnosis) and siblings as controls. Fatigue severity was assessed with the ‘fatigue severity subscale’ of the Checklist Individual Strength (‘CIS-fatigue’). CF was defined as scoring ≥35 on the ‘CIS-fatigue’ and having fatigue symptoms for ≥6 months. Twenty-four parameters were assessed, categorized into assumed fatigue triggering, maintaining and moderating factors. Multivariable logistic regression analyses were carried out to investigate the association of these factors with CF.

**Results:**

A total of 1927 CCS participated in the study (40.7% of invited cohort), of whom 23.6% reported CF (compared with 15.6% in sibling controls, *P* < 0.001). The following factors were associated with CF: obesity [versus healthy weight, odds ratio (OR) 1.93; 95% confidence interval (CI) 1.30-2.87], moderate physical inactivity (versus physical active, OR 2.36; 95% CI 1.67-3.34), poor sleep (yes versus no, OR 2.03; 95% CI 1.54-2.68), (sub)clinical anxiety (yes versus no, OR 1.55; 95% CI 1.10-2.19), (sub)clinical depression (yes versus no, OR 2.07; 95% CI 1.20-3.59), pain (continuous, OR 1.49; 95% CI 1.33-1.66), self-esteem (continuous, OR 0.95; 95% CI 0.92-0.98), helplessness (continuous, OR 1.13; 95% CI 1.08-1.19), social functioning (continuous, OR 0.98; 95% CI 0.97-0.99) and female sex (versus male sex, OR 1.79; 95% CI 1.36-2.37).

**Conclusion:**

CF is a prevalent symptom in CCS that is associated with several assumed maintaining factors, with lifestyle and psychosocial factors being the most prominent. These are modifiable factors and may therefore be beneficial to prevent or reduce CF in CCS.

## Highlights

• Lifestyle factors, e.g., obesity, physical inactivity and poor sleep, are associated with CF.
• Psychosocial factors, e.g., anxiety, depression, self-esteem, helplessness and social functioning, are associated with CF.
• These are modifiable factors and may therefore be beneficial to prevent or reduce CF in CCS.

## Introduction

Chronic fatigue (CF), defined as severe fatigue that persists for at least 6 months, is a common late effect following childhood cancer treatment leading to an impaired quality of life.^1^^,^^2^ Few studies have investigated which factors are associated with CF in childhood cancer survivors (CCS),^3^, ^4^, ^5^, ^6^ but these studies focused on a specific group of factors, e.g., treatment-related factors or demographic factors only, or included small subgroups of CCS participants, limited to certain diagnoses or age groups. Various variables have been associated with fatigue in CCS, including factors related to the childhood cancer (e.g., type of diagnosis or treatment), demographics (e.g., age and sex), and lifestyle and psychosocial aspects (e.g., depression, sleeping disorders, physical (in)activity).^7^, ^8^, ^9^ Due to methodological differences between the studies, however, it is difficult to draw conclusions regarding the strength of the association of these factors with CF in CCS. To investigate the relative relations with CF in CCS, these factors should be studied together in a large cohort of CCS, including all childhood cancer diagnoses.

**Figure 1:**
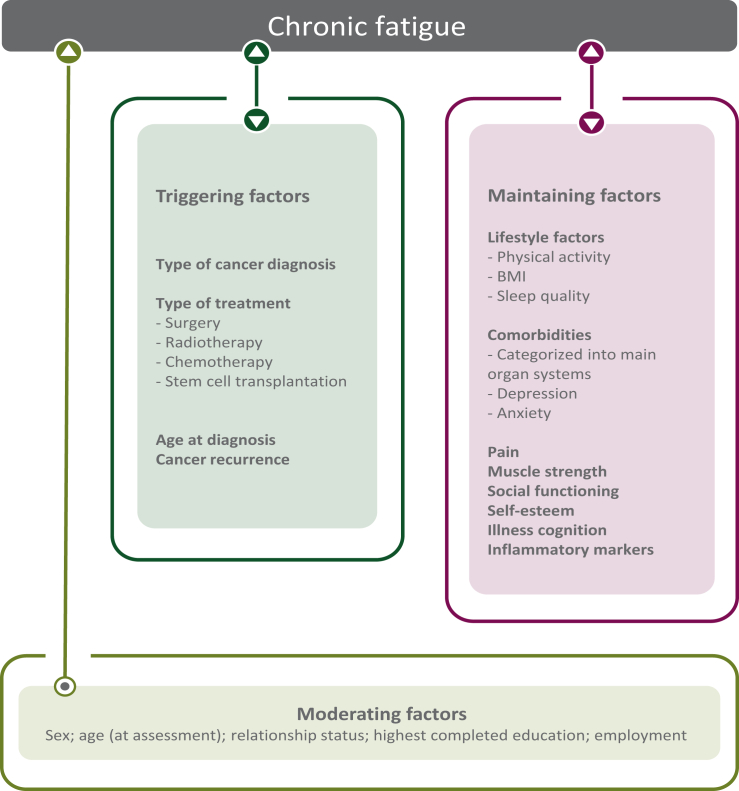
**Proposed model showing assumed relations between factors and CF in CCS.** Figure shows assumed relations of study parameters that have previously been found to be associated with CF.^10^ Triggering factors are assumed to play a role at the onset of fatigue. Maintaining factors are assumed to perpetuate fatigue once triggered. Moderating factors are assumed to have an effect on the strength of fatigue symptoms in individuals. The following change has been made compared with the model presented in^10^: age at diagnosis is considered a triggering factor ensuring all treatment/diagnosis-related factors are categorized in one group of factors as we assume childhood cancer and its treatment to be a triggering factor for fatigue. Also, we believe age at diagnosis to play a part at the onset of fatigue, which is the definition of the assumed triggering factors, and not so much a moderating factor many years after diagnosis. Comorbidities are categorized following previously published main organ system categories.^11^ CF, chronic fatigue; CSS, childhood cancer survivors.

We have proposed a model to arrange factors in one comprehensive multivariable model in order to determine associated factors for CF in CCS.^10^ In the model, factors are categorized based on their assumed relation with CF (Figure 1): triggering factors (thought to play a role at the onset of CF), maintaining factors (thought to perpetuate fatigue once triggered) and moderating factors (might influence the way fatigue expresses in individuals). In a previous questionnaire-based study, where the prevalence of CF was determined in CCS and sibling controls, parts of the proposed model were tested and female sex, being unemployed, having comorbidities and CNS as a childhood cancer diagnosis were associated with CF.^1^ Based on the model performance, however, it was concluded that additional factors need to be considered to explain CF in CCS.^1^ In the current study we collected and analyzed all factors of the proposed model in a large nationwide cohort of CCS, which allowed us to determine the relative association of the factors with CF, in an attempt to address the current knowledge gap. The secondary aim of the study was to confirm previous found prevalence rates of CF in a Dutch nationwide cohort of CCS and sibling controls.^1^

## Methods

### Study design and participants

This study was part of the Dutch Childhood Cancer Survivor Study Late Effect (DCCSS LATER) study part 2.^12^ Participants were included from the DCCSS LATER cohort, a nationwide cohort including all 5-year cancer survivors who were diagnosed before the age of 18 between 1 January 1963 and 31 December 2001 in the Netherlands (*n* = 6165, baseline characteristics described elsewhere^13^). Of this cohort, CCS who were still alive and living in The Netherlands and who were not lost to follow-up or had previously declined to participate in any research were eligible to participate in the study (*n* = 4735).

In addition, siblings of the CCS participants were asked to participate as a control group to compare CF prevalence rates. Contact information was provided by the CCS participants and siblings who had not had cancer who were approached to participate (*n* = 1499).

All participants for the current study were 18 years or older, were able to read and speak Dutch and gave written informed consent to participate. The DCCSS LATER fatigue study was approved by the Medical Research Ethics Committee of the Amsterdam University Medical Centers (registered at toetsingonline.nl, NL34983.018.10).

### Data collection

A detailed description of the methodology and data collection was previously published.^10^ In short, data were collected during a visit at the LATER outpatient clinic, which took place between 2017 and 2020 in one of the seven pediatric oncology centers in the Netherlands. Fatigue severity was assessed with the ‘fatigue severity subscale’ of the Checklist Individual Strength (CIS),^14^ a questionnaire shown to have satisfying psychometric properties in CCS.^15^ CF was defined as a score of 35 or higher on the ‘CIS’ fatigue severity subscale, indicating severe fatigue,^16^ which persists for 6 months or longer (duration of fatigue symptoms was assessed in a separate item next to the CIS). Participants were included if they had sufficient data to determine their fatigue status: at least seven of the eight CIS fatigue severity items completed (with one missing value, the mean of the remaining completed items was imputed) and the duration of fatigue symptoms known (if fatigue severity subscale score ≥35).

Additionally, the following measures were completed as previous research indicated these factors to be related to fatigue^7^, ^8^, ^9^, ^10^: height and weight to calculate body mass index (BMI); social outcomes, e.g., level of education, employment status and relationship status, were assessed using a questionnaire (see Supplementary Table S1, available at https://doi.org/10.1016/j.esmoop.2023.102044 for specific items); somatic comorbidities were assessed using a questionnaire (see Supplementary Table S1, available at https://doi.org/10.1016/j.esmoop.2023.102044 for details) and categorized as having 0, 1-2 or >2 of previously defined physical outcomes^11^; pain was assessed using a six-point Likert scale; physical activity was assessed using the European Prospective Investigation into Cancer and Nutrition (EPIC) physical activity questionnaire and categorized following the four-point physical activity index as being active, moderately active, moderately inactive or inactive^17^^,^^18^; sleep quality was assessed using the Pittsburg Sleep Quality Index (PSQI) with a score of >5 to indicate poor sleep^19^^,^^20^; anxiety and depression were assessed using the Hospital Anxiety and Depression Scale (HADS) with subscale score of ≥8 indicating (sub)clinical anxiety and depression^21^^,^^22^; grip strength was measured with a hand dynamometer to reflect muscle strength^23^; social functioning was assessed using the TNO (Netherlands Organisation for Applied Scientific Research) and AZL (Leiden University Medical Centre) Questionnaire for Adult’s Quality of Life (TAAQOL) social functioning domain^24^; self-esteem was assessed using the Rosenberg Self-Esteem Scale (RSES)^25^^,^^26^; feelings of helplessness, acceptance and perceived benefits were assessed using the Illness Cognition Questionnaire (ICQ)^27^^,^^28^; as an inflammatory marker, C-reactive protein (CRP) levels were analyzed from venous blood samples. Treatment and diagnosis data of primary diagnoses and all recurrences of the CCS participants were collected from medical records by data managers using a uniform protocol.^29^ Details about data collection, categorization and availability for each of these measures are given in Supplementary Table S1, available at https://doi.org/10.1016/j.esmoop.2023.102044. If participants were not able to visit the outpatient clinic, questionnaires could be completed from home digitally.

### Statistical analyses

Differences in baseline characteristics between study participants and non-participants, i.e., non-responders and excluded participants because of missing/insufficient fatigue data or age <18 years, were compared using chi-square tests (with Cramér’s V effect size).

Prevalence rates of CF of CCS and sibling controls were compared using a chi-square analysis and an additional regression analysis to adjust for age and sex differences. To determine which factors were associated with CF in CCS, multivariable logistic regression analyses with CF (yes/no) as dependent variable and the assumed triggering factors (primary childhood cancer diagnosis and treatment, hematopoietic stem cell transplantation, cancer recurrence, age at diagnosis), maintaining factors (BMI, physical activity index, (sub)clinical anxiety, (sub)clinical depression, pain, self-esteem, illness cognition, muscle strength, inflammatory markers, social functioning, sleep problems, comorbidities), and moderating factors (sex, age at assessment, educational level, employment status, relationship status) as independent variables were conducted. Due to power restrictions, we used a forward selection procedure to come to a final model including the most strongly related factors. Firstly, each group of factors—the assumed triggering, maintaining and moderating factors—was analyzed separately in a multivariable model, which ensured the relative associations to be determined, as each analyzed variable was adjusted for the other variables of the same group. Variables that were significantly associated with CF (*P* < 0.05) in the separate models were included in one final multivariable model. Area under the curve (AUC) was calculated for the final model as an indication of model performance, with >0.7 considered acceptable.^30^ Variance inflation factors (VIF) were calculated for all independent variables with a threshold of >5 to test for problematic multicollinearity.^31^

IBM SPSS (IBM Corp. Released 2017. IBM SPSS Statistics for Windows, Version 25.0. Armonk, NY) was used for the analyses. Missing data of the independent variables, assumed to be missing at random (no pattern observed), were imputed with multiple imputation, using the Markov chain Monte Carlo method to create 20 imputed datasets and using Rubin’s rules to pool the analyses.^32^, ^33^, ^34^ Number of missing values per study variable are shown in Supplementary Table S1, available at https://doi.org/10.1016/j.esmoop.2023.102044. All study variables were included in the multiple imputation process, including the diagnosis and treatment-related variables which had no missing values. Complete case analysis was done as a sensitivity analysis.

## Results

### Participant characteristics

A total of 2282 CCS and 506 siblings participated in the DCCSS LATER fatigue study part 2 (48.2% and 33.8% of eligible persons, respectively). Of these participants, 1927 CCS and 449 siblings completed the fatigue questionnaire for the current study (‘CIS fatigue severity’ subscale score and duration fatigue). The flowcharts are depicted in Supplementary Figure S1, available at https://doi.org/10.1016/j.esmoop.2023.102044. Participant characteristics are shown in Table 1.

**Table 1:** Demographic characteristics of CCS & sibling participants and childhood cancer diagnostic and treatment characteristics of the CCS participants

Characteristic	CCS (*n* = 1927)	Siblings (*n* = 449)	*P* value
	*N* (%)	*N* (%)	
Sex			
Male	996 (51.7)	165 (36.7)	<0.001^e^
Female	931 (48.3)	284 (63.3)	
Age at assessment (years)			
Mean (SD)	35.1 (9.3)	36.8 (10.2)	0.001^f^
18-29	599 (31.1)	118 (26.3)	0.023^e^
30-39	737 (38.2)	165 (36.7)	
≥40	591 (30.7)	166 (37.0)	
CF			
Yes	454 (23.6)	70 (15.6)	<0.001^g^
No	1473 (76.4)	379 (84.4)	
Age at diagnosis (years)			
Mean (SD)	6.7 (4.7)		
0-5	886 (46.0)		
>5-10	519 (26.9)		
>10-15	414 (21.5)		
>15-18	108 (5.6)		
Primary childhood cancer diagnosis^a^			
Leukemia	678 (35.3)		
Non-Hodgkin’s lymphoma^b^	234 (12.1)		
Hodgkin’s lymphoma	135 (7.0)		
CNS	177 (9.2)		
Neuroblastoma	111 (5.8)		
Retinoblastoma	10 (0.5)		
Renal tumors	220 (11.4)		
Hepatic tumors	17 (0.9)		
Bone tumors	109 (5.7)		
Soft tissue tumors	141 (7.3)		
Germ cell tumors	65 (3.4)		
Other and unspecified^c^	30 (1.6)		
Period of childhood cancer diagnosis			
1963-1969	29 (1.5)		
1970-1979	255 (13.2)		
1980-1989	607 (31.5)		
>1990	1036 (53.6)		
Childhood cancer treatment^d^			
Surgery only	131 (6.8)		
Chemotherapy, no radiotherapy	1047 (54.3)		
Radiotherapy, no chemotherapy	100 (5.2)		
Radiotherapy and chemotherapy	640 (33.2)		
No treatment/treatment unknown	9 (0.5)		
Stem cell transplantation			
Yes	131 (6.8)		
No	1783 (92.5)		
Unknown	13 (0.7)		
Cancer recurrence			
No	1675 (86.9)		
Yes	252 (13.1)		

CCS, childhood cancer survivors; CF, chronic fatigue; CNS, central nervous system; SD, standard deviation.

a Diagnostic groups included all malignancies covered by the third edition of the International Classification of Childhood Cancer (ICCC-3) as well as multifocal Langerhans cell histiocytosis.

b Includes all morphology codes specified in the ICCC-3 under lymphomas and reticuloendothelial neoplasms, except for Hodgkin’s lymphomas. Also includes multifocal Langerhans cell histiocytosis.

c Includes all morphology codes specified in the ICC-3 under other malignant epithelial neoplasms and malignant melanomas and other and unspecified malignant neoplasms.

d Treatment data included primary treatment and all recurrences.

e Chi-square test.

f Independent *t*-test.

g Chi-square test and logistic regression analysis to correct for age and sex.

Compared with non-participants (non-responders, lacking/missing fatigue questionnaire data or age <18 years*)*, participants were more often female (48.3% versus 39.9%, *P* < 0.001), more often treated with a combination of chemotherapy and radiotherapy (33.2% versus 24.4%, *P* < 0.001) and more often received hematopoietic stem cell transplantation (6.8% versus 3.9%, *P* = 0.001), however effect sizes for these differences were small (0.09, 0.13 and 0.07, respectively). An overview of participant and non-participant characteristics is shown in Supplementary Table S2, available at https://doi.org/10.1016/j.esmoop.2023.102044.

### Prevalence and associated factors

Prevalence of CF was 23.6% in CCS compared with 15.6% in siblings (*P* < 0.001, also after correction for age and sex). Table 2 shows the results of the multivariable logistic regression analyses. Analyses of the separate multivariable models showed no association with the triggering factors, but several maintaining and moderating factors to be associated with CF. The latter were included in the final multivariable model in which obesity [versus healthy weight, odds ratio (OR) 1.93; 95% confidence interval (CI) 1.30-2.87], moderate physical inactivity (versus physical active, OR 2.36; 95% CI 1.67-3.34), poor sleep (yes versus no, OR 2.03; 95% CI 1.54-2.68), (sub)clinical anxiety (yes versus no, OR 1.55; 95% CI 1.10-2.19), (sub)clinical depression (yes versus no, OR 2.07; 95% CI 1.20-3.59), pain (continuous, OR 1.49; 95% CI 1.33-1.66), self-esteem (continuous, OR 0.95; 95% CI 0.92-0.98), helplessness (continuous, OR 1.13; 95% CI 1.08-1.19), social functioning (continuous, OR 0.98; 95% CI 0.97-0.99) and female sex (versus male sex, OR 1.79; 95% CI 1.36-2.37) were found to be associated with CF.

**Table 2:** Results of logistic regression analyses showing lifestyle and psychosocial and demographic factors to be associated with CF

Factor	% Non CF participants∗	% CF participants∗	Separate models^e^		Final model^f^	
	(*n* = 1473)	(*n* = 454)	OR	95% CI	OR	95% CI
**Triggering factors**						
Age at diagnosis (years)						
0-5	47.1	42.3	ref	ref		
>5-10	26.6	28.0	1.15	0.88-1.52		
>10-15	20.6	24.4	1.34	0.99-1.83		
>15-18	5.7	5.3	1.02	0.61-1.72		
Primary childhood cancer diagnosis^a^						
Leukemia	36.3	31.5	ref	ref		
Non-Hodgkin’s lymphoma^b^	12.3	11.7	1.04	0.72-1.51		
Hodgkin’s lymphoma	7.3	6.2	0.76	0.47-1.24		
CNS	8.7	10.8	1.23	0.77-1.96		
Neuroblastoma	5.5	6.6	1.37	0.83-2.28		
Retinoblastoma	0.5	0.7	1.60	0.39-6.66		
Renal tumors	11.3	11.9	1.16	0.80-1.69		
Hepatic tumors	1.1	0.2	0.26	0.03-2.00		
Bone tumors	5.4	6.6	1.25	0.77-2.03		
Soft tissue tumors	6.8	9.0	1.37	0.90-2.10		
Germ cell tumors	3.5	2.9	0.87	0.45-1.68		
Other and unspecified^c^	1.4	2.0	1.38	0.59-3.23		
Childhood cancer treatment^d^						
Surgery only	6.5	7.7	ref	ref		
Chemotherapy, no radiotherapy	56.2	48.2	0.88	0.54-1.46		
Radiotherapy, no chemotherapy	4.9	6.2	1.12	0.62-2.05		
Radiotherapy and chemotherapy	31.8	37.7	1.25	0.76-2.05		
No treatment/treatment unknown	0.5	0.2	0.37	0.05-3.12		
Hematopoietic stem cell transplantation						
No	92.0	94.3	ref	ref		
Autologous	2.4	2.6	1.14	0.57-2.28		
Allogeneic	4.8	2.9	0.57	0.30-1.08		
Unknown	0.8	0.2	0.23	0.03-1.81		
Recurrence						
No	86.6	87.9	ref	ref		
Yes	13.4	12.1	0.81	0.57-1.14		
**Maintaining factors**						
BMI						
Healthy weight	55.2	44.7	ref	ref	ref	ref
Underweight	2.7	4.0	1.26	0.56-2.85	1.36	0.61-3.04
Overweight	31.9	31.4	1.30	0.95-1.77	1.30	0.95-1.78
Obese	10.2	19.9	**2.03**	**1.37-3.02**	**1.93**	**1.30-2.87**
Physical activity index						
Inactive	4.6	10.1	1.30	0.71-2.38	1.15	0.62-2.15
Moderately inactive	20.3	31.7	**2.42**	**1.72-3.40**	**2.36**	**1.67-3.34**
Moderately active	23.8	21.9	1.42	0.98-2.06	1.36	0.94-1.97
Active	51.3	36.3	ref	ref	ref	ref
HADS						
(Sub)clinical anxiety (no = ref)	13.4	43.3	**1.53**	**1.09-2.15**	**1.55**	**1.10-2.19**
(Sub)clinical Depression (no = ref)	3.2	22.4	**2.01**	**1.15-3.51**	**2.07**	**1.20-3.59**
Pain						
Total score, 1-6 Likert scale	1.74	2.74	**1.51**	**1.35-1.69**	**1.49**	**1.33-1.66**
Self-esteem						
RSES total score (continuous)	33.6	28.7	**0.94**	**0.90-0.97**	**0.95**	**0.92-0.98**
Illness cognition (continuous)						
Helplessness total score	7.4	10.3	**1.11**	**1.05-1.17**	**1.13**	**1.08-1.19**
Acceptance total score	20.3	17.9	0.98	0.94-1.02		
Disease benefits total score	16.9	16.1	1.02	0.99-1.06		
Muscle strength (continuous)						
Handgrip strength in kg	40.7	36.1	0.99	0.98-1.00		
Inflammatory markers						
CRP in mg/l (continuous)	4.4	5.4	1.01	0.99-1.03		
Social functioning (continuous)						
TAAQOL subscale score	89.6	74.4	**0.98**	**0.97-0.99**	**0.98**	**0.97-0.99**
PSQI						
Poor sleeper (no = ref)	28.3	64.0	**2.06**	**1.56-2.72**	**2.03**	**1.54-2.68**
Comorbidities						
0	48.0	33.7	ref			
1-2	44.1	47.0	1.09	0.81-1.45		
>2	7.9	19.3	1.31	0.83-2.08		
**Moderating factors**						
Sex						
Male	56.5	36.1	ref	ref	ref	ref
Female	43.5	63.9	**2.29**	**1.83-2.87**	**1.79**	**1.36-2.37**
Age at assessment (years)						
18-29	32.9	25.1	ref	ref	ref	ref
30-39	38.1	38.8	**1.67**	**1.26-2.22**	1.25	0.89-1.76
≥40	29.0	36.1	**1.91**	**1.42-2.56**	1.23	0.86-1.77
Educational level						
Low	12.4	16.4	ref	ref		
Middle	41.9	46.7	0.93	0.66-1.31		
High	45.7	36.9	0.74	0.52-1.04		
Employment status						
Employed	88.8	72.3	ref	ref	ref	ref
Not employed	11.2	27.7	**2.79**	**2.10-3.72**	1.34	0.92-1.95
Relationship status						
In a relationship	77.8	73.0	**0.72**	**0.54-0.97**	0.95	0.67-1.34
Not in a relationship	22.2	27.0	ref	ref	ref	ref

Values in bold indicate statistically significant associations.

95% CI, 95% confidence interval; BMI, body mass index; CF, chronic fatigue; CNS, central nervous system; CRP, C-reactive protein; HADS, Hospital Anxiety and Depression Scale; PSQI, Pittsburg Sleep Quality Index; RSES, Rosenberg Self-Esteem Scale; TAAQOL, TNO (Netherlands Organisation for Applied Scientific Research) and AZL (Leiden University Medical Centre) Questionnaire for Adult’s Quality of Life.

a Diagnostic groups included all malignancies covered by the third edition of the International Classification of Childhood Cancer (ICCC-3) as well as multifocal Langerhans cell histiocytosis.

b Includes all morphology codes specified in the ICCC-3 under lymphomas and reticuloendothelial neoplasms, except for Hodgkin’s lymphomas. Also includes multifocal Langerhans cell histiocytosis.

c Includes all morphology codes specified in the ICC-3 under other malignant epithelial neoplasms and malignant melanomas and other and unspecified malignant neoplasms.

d Treatment data included primary treatment and all recurrences.

e Three separate multivariable logistic regression models with chronic fatigue as dependent variable and the assumed triggering, maintaining and moderating factors as independent variables. Each variable was adjusted for the other variables of the same group.

f Chronic fatigue as dependent variable and the statistically significant (*P* < 0.05) factors from the separate models as independent variables in one ‘final model’. Each variable was adjusted for the other variables included in this final model.

∗ Mean scores are shown for continuous variables.

**Figure 2:**
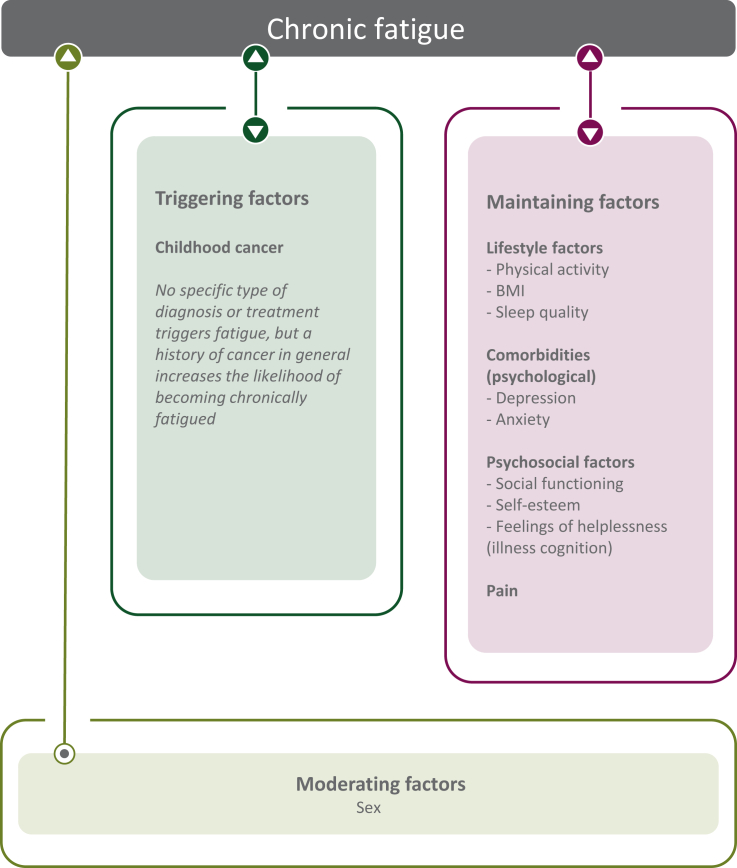
**Adjusted model showing CF-associated factors in CCS.** Figure shows the factors that were statistically significantly associated with CF in the final model. Triggering factors are assumed to play a role at the onset of fatigue. No specific diagnosis or treatment was found to be associated with CF, still the prevalence of CF in CCS was increased compared with sibling controls (23.6% versus 15.6%), suggesting that a history of cancer in general plays a role in triggering symptoms of fatigue. Maintaining factors are assumed to perpetuate fatigue once triggered. Moderating factors are assumed to have an effect on the strength of fatigue symptoms in individuals. CF, chronic fatigue; CSS, childhood cancer survivors.

AUC of this model was >0.86 for each imputed dataset (20 imputations, pooled AUC could not be generated), indicating excellent model performance. VIF were <2.0 for all factors included in the analyses, suggesting no problematic multicollinearity to be present. Based on the found associations, the proposed model that was presented in Figure 1 was adjusted and now only includes the factors that were found to be statistically significantly associated with CF (Figure 2). Results of a *post hoc* analysis, investigating in more detail the relation between CF and number of comorbidities, are shown in Supplementary Table S3, available at https://doi.org/10.1016/j.esmoop.2023.102044.

Complete case analysis showed the same variables to be associated with CF (see Supplementary Table S4, available at https://doi.org/10.1016/j.esmoop.2023.102044), except for depression. The complete case analysis lacks statistical power, however, therefore reliability of these results is questionable.

## Discussion

In the current study we present the prevalence rate and associated factors of CF in a nationwide cohort of CCS. Results showed various assumed maintaining factors to be associated with CF: lifestyle factors—e.g., physical inactivity, obesity and poor sleep—, psychosocial factors—e.g., anxiety, depression, self-esteem, social functioning and feelings of helplessness—and pain showed the most strong associations with CF.

### CF in CCS

We showed that approximately one in four CCS have CF, emphasizing its magnitude in this population and replicating previous findings.^1^ The increased prevalence of CF in CSS compared with sibling controls (23.6% versus 15.6%) suggests the experience of having had cancer during childhood to increase the likelihood of becoming chronically fatigued. It could be that symptoms of fatigue persist from the period of childhood diagnosis, where fatigue is an often seen as a side-effect of cancer and its treatment,^35^^,^^36^ but fatigue might also manifest at a later stage in life. In the latter case, it may be due to the cancer and its treatment that CCS are more prone to develop CF over time. Only prospective studies, however, can inform us on how CF develops over time.

No association between CF and diagnosis and treatment-related factors was found. This suggests that not a particular type of diagnosis or treatment triggers CF, but a history of cancer in general (thus explaining the increased prevalence in CCS compared with sibling controls). In previous studies, relations between CF and specific diagnosis-related factors have been found, with an association of CF with CNS as a childhood cancer diagnosis being the most illustrative.^1^^,^^37^ It was hypothesized that, as a result of treatment to the head/cranium, these CCS are at increased risk for developing fatigue, similar as they are at risk for neurocognitive impairment.^4^^,^^38^ Results showed no such association to be present, however, when assessed in a large cohort including all childhood diagnoses. Also, no significant association was found between CF and radiotherapy locations involving the head/cranium, i.e., head/cranium, spinal or total body irradiation (univariable analyses, data not shown). Results are in line with literature showing CNS tumor patients treated with cranial/spinal irradiation to have normalized levels of fatigue after treatment completion compared with pre-treatment.^39^

Lifestyle and psychosocial factors were found to be associated with CF. Lifestyle and psychosocial factors are potentially modifiable factors, in contrast to disease and treatment-related factors. Therefore, focusing on these modifiable factors for prevention or tailored interventions might be beneficial to reduce CF. The recommendation guideline for the surveillance of fatigue in childhood, adolescent and young adult (CAYA) survivors, proposed by the International Guideline Harmonization Group (IGHG), stated that potential risk factors for fatigue are clinical, e.g., psychological distress, health issues or pain, and demographical factors, e.g., age, sex, employment and education, not diagnosis or treatment-related factors.^9^ Evidence to support these findings was low to moderate, however, mainly because of the lack of studies using a validated fatigue measure.^9^ The current study, using a validated fatigue measure,^15^ confirms that not diagnosis and treatment-related factors, but lifestyle and psychosocial factors are associated with CF in CCS. Our results are in concordance with studies in other patient populations suggesting that CF-related factors are not disease specific, i.e., diagnosis or treatment-related, but are trans-diagnostic, i.e., are similar for different long-term medical conditions, such as lifestyle and psychosocial-related factors.^40^, ^41^, ^42^ These studies found that factors such as female sex, physical inactivity, sleep disturbances, depression and pain, which were found to be associated with CF in our study as well, were associated with fatigue across different (chronic) diseases and to a same extent in healthy subjects. This suggests that fatigue is a generic symptom which expresses similarly over different (patient) populations, and presumably asks for a generic approach.

### Clinical implications

We found that several assumed maintaining factors, i.e., psychosocial and lifestyle factors, were associated with CF. Therefore, when CCS present with fatigue symptoms, it might be good to screen for these associated factors or discuss them during consultation. Symptoms tend to cluster, as was shown in cancer patients and survivors of adult-onset cancer,^43^, ^44^, ^45^ therefore it is likely for CSS to present with multiple symptoms simultaneously as well.

In addition, psychosocial and lifestyle factors are assumed modifiable variables and are therefore potentially interesting to target when aiming to reduce CF. For example, previous studies have shown psychological interventions, e.g., cognitive behavioral therapy (CBT), to be effective interventions to reduce fatigue levels in survivors of adult-onset cancer and also a pilot study with CBT in CCS showed promising results.^46^, ^47^, ^48^, ^49^ Also, physical activity interventions, e.g., lifestyle and exercise counseling and exercise or yoga programs, show promising results.^50^^,^^51^ Both psychological and physical activity interventions are recommended by the American Society of Clinical Oncology guideline to treat fatigue in survivors of adult-onset cancer^52^ and the IGHG recommendations for fatigue-surveillance in CAYA survivors.^9^ The current results show that targeting psychological and/or lifestyle factors might indeed be beneficial to reduce fatigue in CCS, thus encouraging a similar recommendation to treat CF in CCS as well. To determine the effect of CBT and physical activity interventions in CCS, however, studies in larger sample sizes and regional/cultural specific populations are needed to confirm and validate these results before vast recommendations can be made. Next to possible interventions that tackle CF-related issues, prevention strategies might benefit from focusing on CF-associated factors, such as lifestyle factors, as they might reduce the risk of developing CF.

### The role of comorbidities

Previous literature showed that having one or more comorbidities was associated with fatigue in cancer survivors.^1^^,^^53^^,^^54^ In the current study, having (multiple) comorbidities was not associated with CF. *Post hoc* analysis did show a univariable relation between having comorbidities and CF, however, suggesting other factors to mediate this relation. Reduced physical activity, sleep problems, pain, lower self-esteem, helplessness and problems with social functioning were found as possible mediators (Supplementary Table S3, available at https://doi.org/10.1016/j.esmoop.2023.102044). A plausible pathway explaining the relation between number of comorbidities and CF could therefore be that having one or multiple comorbidities negatively influences other factors such as pain, helplessness, self-esteem, social functioning, physical activity and sleep quality, which causes these patients to experience more fatigue. As CCS are at increased risk for various health issues,^55^^,^^56^ this hypothesis might also partly explain the increased prevalence of CF in CCS.

### Strengths and limitations

This study is part of a nationwide collaboration and includes a large nationwide cohort consisting of all 5-year survivors who were diagnosed between 1963 and 2002 including all childhood malignancies, which contributes to the generalizability of the results. Being one of 16 sub-studies of the DCCSS LATER study^12^ ensured a lot of topics to be studied at the same time in the same cohort. This unique study design made it possible to include many factors that were hypothesized to be associated with CF in CCS,^10^ which ensured these factors to be analyzed relative to each other, resulting in a more complete picture of CF and its associated factors. The high AUC of the model (>0.86 in all imputed datasets), which can be interpreted as a proxy for the completeness of the model, also reflects this as it shows excellent performance of the final model.^30^ Compared with the previously conducted questionnaire-based study where only part of the proposed model was tested,^1^ the current model shows improved model performance (0.86 versus 0.71 in previous study), suggesting the current model to be more complete.

No information was available on current smoking habits or alcohol consumption, which is considered a limitation. Although current literature shows smoking is not associated with fatigue,^1^^,^^9^ and therefore including it in the model would probably not have affected the results, information on alcohol consumption could have been of added value to the model. Another limitation is that we cannot discard the possibility of selection bias, as small differences between participants and non-participants were seen. Therefore, it is possible that certain subgroups of CCS were less/more inclined to participate in the current study. Effect sizes for differences between participants and non-participants were small, however, therefore it is unlikely for these differences to have impacted the results of the study.

Lastly, we elaborated on assumed triggering, maintaining and moderating factors of CF. As data were cross-sectional, however, no causal inferences can be made based on these findings. The exact relation between factors needs to be confirmed in a longitudinal study.

## Conclusion

CF is a prevalent symptom in CCS that is associated with several assumed maintaining factors, with lifestyle and psychosocial factors being the most prominent. These are modifiable factors and may therefore be beneficial to prevent or reduce CF in CCS.
