# Supplementary material for: Chronic fatigue in childhood cancer survivors is associated with lifestyle and psychosocial factors; a DCCSS LATER study
Source: ESMO Open. 2023 Nov 2;8(6):102044. doi: 10.1016/j.esmoop.2023.102044 (PMC10774970; doi:10.1016/j.esmoop.2023.102044)
Supplement: Supplementary Tables [file mmc1.docx]

Supplementary material.

**Supplementary Figure 1. Flowcharts of CCS and sibling study participants**

**
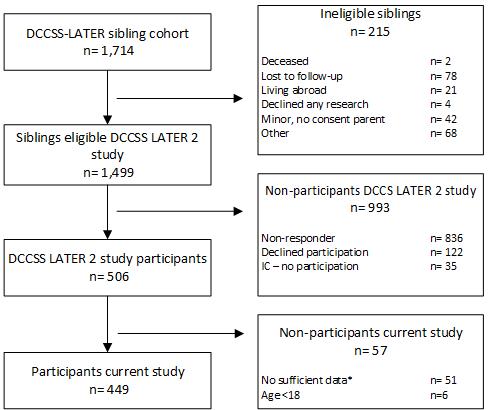
**

**
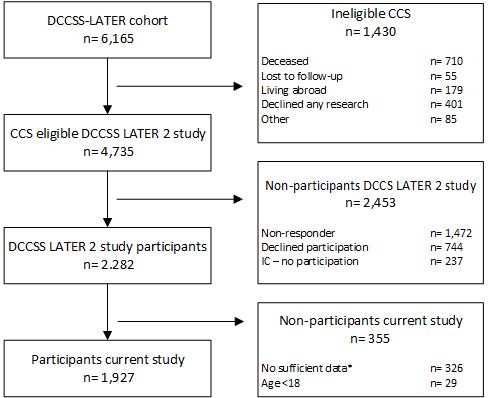
**

**Sufficient data to determine fatigue status: at least 7 of the 8 CIS fatigue severity items completed (with one missing value, the mean of the remaining completed items was imputed) + duration of fatigue symptoms completed (if fatigue severity subscale score ≥35).*

**Supplementary Table 1. Overview of data collection, -categories and -availability CCS participants (n=1927).**

| **Variable of interest** | **Type of questionnaire or questionnaire item (when applicable)** | **Categories** | **Data availability (% missing)** |
| --- | --- | --- | --- |
| Age at assessment | Date of assessment – date of birth | In years (continuous) | n=1927 |
| BMI | Length (cm) and weight (kg) measured during clinic visit. BMI was calculated using the formula: kg/m^2^ | Underweight: BMI <18.5  Healthy weight: BMI between 18.5 and 24.99  Overweight: BMI between 25 and 29.99  Obesity: BMI ≥30 | n=1865 (3%) |
| Employment status | Do you currently have work? Yes/no | Employed: Currently employed  Unemployed: Currently unemployed | n=1807 (6%) |
| Educational level | What is the highest level of education you have completed? Answer options: primary education, vocational education, preparatory secondary vocational education, secondary vocational education, school of higher general secondary education, pre-university education, higher vocational education, university, special school. | Low: Primary education, vocational education, special school  Middle: Preparatory secondary vocational education, secondary vocational education, school of higher general secondary education, pre-university education  High: Higher vocational education, university | n=1809 (6%) |
| Relationship status | Are you currently in a relationship? Yes/no | Partner: Currently in relationship  No partner: Currently not in a relationship | n=1610 (16%) |
| Somatic comorbidities | Could you please indicate if you currently or ever did suffer any of the conditions stated below. If so, could you please state in what year you got the diagnosis and if you are currently using medication for it (and if so, which medication).  The following conditions were listed: heart attack, angina pectoris, heart valve defect, pericarditis, cardiomyopathy, heart failure, cardiac arrhythmia, heart defect since birth ….*, other heart disease…*, stroke, vascular abnormality ….*, condition with increased risk for thrombosis (protein C deficiency, protein S deficiency, factor V Leiden mutation, other ….*), hypertension, high cholesterol, stomach or intestine problems, lung disease ….*, kidney problems (for example kidney stones, too much protein in your urine, cysts) ….*, adrenal glands problems ….*, liver problems ….*, musculoskeletal problems (for example arm/leg/elbow/knee) ….*, diabetes, epilepsy, cataract, tinnitus, reduced height growth, hypothyroidism, hyperthyroidism, thyroid nodule, other thyroid conditions ….*, other condition related to hormone regulation ….*, other condition ….*  Additional items assessing problems with the respiratory system and hearing loss were completed. | Participants were categorized as having 0, 1-2 or >2 of the following comorbidities, as described by Streefkerk et al. [11] ^a^:  Neoplasms,  Cardiac-,  Vascular-,  Respiratory-,  Gastro-intestinal-,  Hepatobiliary-,  Renal and urinary tract-,  Endocrine-,  Musculoskeletal-,  Ear-,  Eye-,  Nervous system-,  Other conditions. | n=1912 (<1%)  n=1902 (1%)  n=1896 (2%)  n=1902 (1%)  n=1758 (9%)  n=1904 (1%)  n=1902 (1%)  n=1888 (2%)  n-1905 (1%)  n=1909 (<1%)  n=1903 (1%)  n=1907 (1%)  n=1908 (<1%) |
| Pain | How much pain did you experience in the past four weeks? Answer options: none (1), very mild, mild, some, much, very much (6). | Total pain score (range 1-6) | n=1883 (2%) |
| Physical activity | EPIC physical activity questionnaire items were used to categorize participants using the four-point physical activity index as proposed by Wareham et al. [17]. | Inactive: sedentary job and no recreational activity  Moderately inactive: sedentary job with <0.5h recreational activity per day or standing job with no recreational activity  Moderately active: sedentary job with 0.5-1h recreational activity per day or physical job with no recreational activity  Active: sedentary job with >1h recreational activity per day or standing job with >0.5h recreational activity per day or physical job with at least some recreational activity or heavy manual job.  Recreational activity was defined as activities of MET (Metabolic Equivalent) scores ≥4, as described in the compendium of physical activities (this includes biking and doing sports activities) [18]. | n=1744 (9%) |
| Anxiety | The outcomes of the seven items of the HADS anxiety subscale were added up and the total score was used to indicate person’s as having anxiety yes/no. | No anxiety: HADS anxiety subscale score <8  Anxiety: HADS anxiety subscale score ≥8 | n=1624 (16%) |
| Depression | The outcomes of the seven items of the HADS depression subscale were added up and the total score was used to indicate person’s as having depression yes/no. | No depression: HADS depression subscale score <8  Depression: HADS depression subscale score ≥8 | n=1622 (16%) |
| Muscle strength | Grip strength was measured four times (two times left arm, two times right arm). The mean score was used to indicate muscle strength. Measurements were done in seated position, with the upper arm next to the body and the elbow flexed 90 degrees. | Mean grip strength in kg (continuous) | n=1543 (20%) |
| Social functioning | TAAQOL social functioning domain items were added up and linearly transformed to a 0-100 scale following instructions described elsewhere [24] (higher scores reflecting better social functioning) | TAAQOL social functioning domain score (continuous) | n=1679 (13%) |
| Self-esteem | Ten items of the RSES were added up (total score ranging 10-40), with a higher score reflecting higher self-esteem | RSES total score (continuous) | n=1633 (15%) |
| Illness cognition | The three subscales of the ICQ (helplessness, acceptance and perceived benefits) were calculated by adding up the six items of each subscale (subscale scores ranging 6-24). Higher scores indicate more helplessness, acceptance and perceived benefits respectively. | ICQ subscale helplessness (continuous)  ICQ subscale acceptance (continuous)  ICQ subscale perceived benefits (continuous) | n=1601 (17%)  n=1593 (17%)  n=1597 (17%) |
| Sleep quality | Seven PSQI component scores and total scores were calculated using scoring instructions described elsewhere Buysse et al.[19]. To compute the global score, the 7 component scores were added up. If at least 5 of the 7 component scores were present, a mean of the non-missing components was used to impute the missing value(s), as proposed by Beck et al. [20] | Good sleeper: PSQI total score ≤5  Poor sleeper: PSQI total score >5 | n=1831 (5%) |
| Inflammatory marker | CRP levels were measured in venous blood samples that were drawn after overnight fasting and stored at -80 °C. CRP levels <0.6 mg/L were listed as missing. | CRP level (mg/L) | n=1420 (26%) |

**Participants could, if applicable, write the name of a condition at the dashed line. Abbreviations: BMI=Body Mass Index; EPIC=European Prospective Investigation into Cancer and Nutrition; HADS=**Hospital Anxiety and Depression Scale; TAAQOL=* *TNO (Netherlands Organisation for Applied Scientific Research) and AZL (Leiden University Medical Centre) Questionnaire for Adult’s Quality of Life; RSES= Rosenberg Self-Esteem Scale; ICQ= Illness Cognition Questionnaire; PSQI=**Pittsburg Sleep Quality Index; CRP= C-Reactive Protein. ^a^ Compared to the conditions listed by Streefkerk et al. [11], the following conditions differ slightly: Eye conditions include only cataract and eye removal and for ear conditions only hearing loss was assessed.* *The following conditions were not included: urinary tract obstruction, underweight, obesity, headache, hydrocephalus, other neurological conditions, decreased pulmonary functioning, dermatological conditions.*

| **Characteristic** | **Participants**  **(n=1927)** | **Non participants (n=2064) *** | **P-value ^e^** | **ES ^f^** |
| --- | --- | --- | --- | --- |
|  | **N (%)** | **N (%)** |  |  |
| Sex  Male  Female | 996 (51.7)  931 (48.3) | 1241 (60.1)  823 (39.9) | <0.001 | 0.09 |
| Year of birth  <1960  1960 – 1969  1970 – 1979  1980 – 1989  ≥1990 | 21 (1.1)  152 (7.9)  502 (26.1)  740 (38.4)  512 (26.5) | 21 (1.0)  151 (7.3)  516 (25.0)  789 (38.2)  587 (28.5) | 0.11 | 0.05 |
| Age at diagnosis (years)  0-5  5-10  10-15  15-18 | 886 (46.0)  519 (26.9)  414 (21.5)  108 (5.6) | 977 (47.3)  564 (27.3)  393 (19.1)  130 (6.3) | 0.24 | 0.03 |
| Primary childhood cancer diagnosis ^a^  Leukemia  Non-Hodgkin lymphoma ^b^  Hodgkin lymphoma  CNS  Neuroblastoma  Retinoblastoma  Renal tumors  Hepatic tumors  Bone tumors  Soft tissue tumors  Germ cell tumors  Other and unspecified ^c^ | 678 (35.2)  234 (12.1)  135 (7.0)  177 (9.2)  111 (5.8)  10 (0.5)  220 (11.4)  17 (0.9)  109 (5.7)  141 (7.3)  65 (3.4)  30 (1.6) | 684 (33.1)  237 (11.5)  148 (7.2)  244 (11.8)  108 (5.2)  14 (0.7)  224 (10.9)  25 (1.2)  112 (5.4)  152 (7.4)  86 (4.2)  30 (1.5) | 0.35 | 0.06 |
| Period of childhood cancer diagnosis  1963-1969  1970-1979  1980-1989  >1990 | 29 (1.5)  255 (13.2)  607 (31.5)  1036 (53.8) | 18 (0.9)  255 (12.4)  631 (30.6)  1160 (53.1) | 0.15 | 0.04 |
| Childhood cancer treatment ^d^  Surgery only  Chemotherapy, no radiotherapy  Radiotherapy, no chemotherapy  Radiotherapy and chemotherapy  No treatment/treatment unknown | 131 (6.8)  1047 (54.3)  100 (5.2)  640 (33.2)  9 (0.5) | 244 (11.8)  1163 (56.3)  125 (6.1)  503 (24.4)  23 (1.1) | <0.001 | 0.13 |
| Hematopoietic stem cell transplantation  Yes  No  Missing | 131 (6.8)  1783 (92.5)  13 (0.7) | 81 (3.9)  1974 (95.6)  9 (0.5) | 0.001 | 0.07 |
| Cancer recurrence  No  Yes | 1675 (86.9)  252 (13.1) | 1821 (88.2)  243 (11.8) |  | 0.02 |

**Supplementary Table 2. Comparison CCS participants vs. non-participants.**

*Abbreviations: CCS= Childhood Cancer Survivors; CNS=Central Nervous System; ES=Effect Size*

**Non-participants were invited to participate but did not return or complete the fatigue questionnaire (non-responders + lacking/missing complete or fatigue specific questionnaire data). In flowchart in Supplementary Figure 1, non-participants DCCSS LATER 2 (n=2.453) and non-participants current study (n=355) are added up, minus 744 CCS who declined participation and who were therefore not analyzed.*

*^a^ Diagnostic groups included all malignancies covered by the third edition of the International Classification of Childhood Cancer (ICCC-3) as well as multifocal Langerhans cell histiocytosis.*

*^b^ Includes all morphology codes specified in the ICCC-3 under lymphomas and reticuloendothelial neoplasms, except for Hodgkin lymphomas. Also includes multifocal Langerhans cell histiocytosis.*

*^c^ Includes all morphology codes specified in the ICC-3 under other malignant epithelial neoplasms and malignant melanomas and other and unspecified malignant neoplasms.*

*^d^ Treatment data included primary treatment and all recurrences.*

*^e^ Chi-Square test*

*^f^ Cramér’s V effect size (<0.1=little, 0.1=low, 0.3=medium, 0.5=high).*

**Supplementary Table 3. Post-hoc analyses showing relation between number of comorbidities and chronic fatigue, before and after adding other factors to the model.**

|  | ***Starting model*** | ***Starting model***  ***+physical activity*** | ***Starting model***  ***+pain*** | ***Starting model***  ***+anxiety*** | ***Starting model***  ***+depression*** | ***Starting model***  ***+BMI*** | ***Starting model***  ***+sleep***  ***problems*** |
| --- | --- | --- | --- | --- | --- | --- | --- |
|  | ***OR (95%CI)*** | ***OR (95%CI)*** | ***OR (95%CI)*** | ***OR (95%CI)*** | ***OR (95%CI)*** | ***OR (95%CI)*** | ***OR (95%CI)*** |
| **Number of Comorbidities**  0  1-2  >2 | ref  1.32 (1.03 – 1.68)  2.82 (1.98 – 4.00) | ref  1.28 (1.00 – 1.64)  2.54 (1.78 – 3.65) | ref  1.20 (0.93 – 1.55)  2.27 (1.55 – 3.30) | ref  1.31 (1.02 – 1.70)  2.58 (1.79 – 3.74) | ref  1.29 (0.99 – 1.66)  2.65 (1.81 – 3.86) | ref  1.31 (1.02 – 1.67)  2.78 (1.94 – 3.96) | ref  1.34 (1.04 – 1.73)  2.54 (1.75 – 3.68) |
|  | ***Starting model***  **+muscle**  **strength** | ***Starting model***  **+social functioning** | ***Starting model***  **+self-**  **esteem** | ***Starting model***  **+helplessness** | ***Starting model***  **+acceptance** | ***Starting model***  **+disease benefits** | ***Starting model***  **+CRP** |
|  | **OR (95%CI)** | **OR (95%CI)** | **OR (95%CI)** | **OR (95%CI)** | **OR (95%CI)** | **OR (95%CI)** | **OR (95%CI)** |
| **Number of Comorbidities**  0  1-2  >2 | ref  1.29 (1.01 – 1.65)  2.68 (1.88 – 3.81) | ref  1.29 (0.99 – 1.68)  2.37 (1.62 – 3.47) | ref  1.27 (0.97 – 1.65)  2.45 (1.67 – 3.60) | ref  1.06 (0.82 – 1.38)  1.46 (0.97 – 2.21) | ref  1.34 (1.04 – 1.73)  2.62 (1.82 – 3.78) | ref  1.37 (1.07 – 1.75)  2.97 (2.08 – 4.23) | ref  1.31 (1.03 – 1.68)  2.75 (1.93 – 3.92) |

*Results of logistic regression analyses are shown with chronic fatigue as dependent variable and number of comorbidities (combined with each hypothesized mediating factor) as independent variables. Starting model is univariable logistic regression model with CF as dependent variable and number of comorbidities as independent variable (adjusted for age and sex). Every hypothesized mediating factor is added to the starting model to determine the change in OR. A change of >10% in OR compared to the starting model indicates that particular factor to be a possible mediator/confounder for the association between number of comorbidities and chronic fatigue (this was the case for the factors physical activity, pain, sleep problems, social functioning, self-esteem and helplessness). All models were adjusted for age and sex.*

**Supplementary Table 4. Results of the logistic regression analyses with complete cases only, n=775 (sensitivity analysis)**

| Factor | % non CF participants* | % CF  participants* | Separate models ^e^ | | Final model ^f^ | |
| --- | --- | --- | --- | --- | --- | --- |
|  | (n=581) | (n=194) | OR | 95% CI | OR | 95% CI |
| **Triggering factors** |  |  |  |  |  |  |
| Age at diagnosis (years)  0-5  >5-10  >10-15  >15-18 | 46.5  27.9  19.8  5.9 | 45.4  26.3  25.3  3.1 | ref  1.03  1.45  0.55 | ref  0.68 – 1.57  0.90 – 2.34  0.21 – 1.46 |  |  |
| Primary childhood cancer diagnosis ^a^  Leukemia  Non-Hodgkin lymphoma ^b^  Hodgkin lymphoma  CNS  Neuroblastoma  Retinoblastoma  Renal tumors  Hepatic tumors  Bone tumors  Soft tissue tumors  Germ cell tumors  Other and unspecified ^c^ | 37.9  13.9  8.1  7.4  4.6  0.3  11.0  1.4  4.5  6.4  3.4  1.0 | 34.0  10.8  6.2  9.3  6.2  1.0  12.4  0.0  5.7  9.8  3.1  1.5 | ref  0.80  0.66  1.10  1.39  2.93  1.15  n/a  1.33  1.59  0.88  1.54 | ref  0.45 – 1.42  0.31 – 1.43  0.49 – 2.43  0.62 – 3.14  0.35 – 24.7  0.65 – 2.03  n/a  0.60 – 2.96  0.83 – 3.05  0.32 – 2.39  0.35 – 6.78 |  |  |
| Childhood cancer treatment ^d^  Surgery only  Chemotherapy, no radiotherapy  Radiotherapy, no chemotherapy  Radiotherapy and chemotherapy  No treatment/treatment unknown | 5.0  62.0  3.4  29.1  0.5 | 6.2  54.1  5,7  33.5  0.5 | ref  0.93  1.57  1.40  0.86 | ref  0.39 – 2.22  0.55 – 4.47  0.59 – 3.32  0.08 – 9.37 |  |  |
| Hematopoietic stem cell transplantation  No  Autologous  Allogeneic  Unknown | 90.9  2.4  6.0  0.7 | 94.3  2.6  3.1  0.0 | ref  1.19  0.45  n/a | ref  0.39 – 3.65  0.55 – 4.47  n/a |  |  |
| Recurrence  No  Yes | 86.4  13.6 | 89.2  10.8 | ref  0.63 | ref  0.35 – 1.12 |  |  |
| **Maintaining factors** |  |  |  |  |  |  |
| BMI  Healthy weight  Underweight  Overweight  Obese | 56.3  1.5  31.8  10.3 | 42.8  2.1  34.5  20.6 | ref  0.59  1.49  **2.10** | ref  0.10 – 3.29  0.94 – 2.36  **1.17 – 3.78** | ref  0.56  1.49  **2.13** | ref  0.10 – 3.07  0.94 – 2.37  **1.18 – 3.85** |
| Physical activity index  Inactive  Moderately inactive  Moderately active  Active | 3.6  21.5  23.1  51.8 | 9.8  30.4  23.7  36.1 | 1.55  **2.21**  **1.82**  ref | 0.60 – 3.96  **1.33 – 3.67**  **1.09 – 3.06**  ref | 1.78  **2.04**  **1.73**  ref | 0.72 – 4.52  **1.23 – 3.39**  **1.18 – 1.66**  ref |
| HADS  (Sub)clinical Anxiety (no = ref)  (Sub)clinical Depression (no=ref) | 13.3  3.1 | 46.9  22.7 | **1.67**  2.09 | **1.00 – 2.76**  0.82 – 3.43 | **1.74** | **1.18 – 1.66** |
| Pain  Total score, 1-6 Likert scale | 1.76 | 2.70 | **1.43** | **1.21 – 1.70** | **1.40** | **1.33 – 1.66** |
| Self-esteem  RSES total score (continuous) | 34.2 | 29.0 | **0.94** | **0.89 – 0.99** | **0.93** | **0.89 – 0.98** |
| Illness Cognition (continuous)  Helplessness total score  Acceptance total score  Disease benefits total score | 7.1  20.5  17.3 | 9.8  18.3  16.4 | **1.09**  0.99  1.02 | **1.00 – 1.18**  0.93 – 1.06  0.97 – 1.07 | **1.11** | **1.03 – 1.20** |
| Muscle strength (continuous)  Handgrip strength in kg | 40.7 | 35.1 | **0.98** | **0.96 – 1.00** | 1.00 | 0.98 – 1.04 |
| Inflammatory markers  CRP in mg/L (continuous) | 3.4 | 5.2 | 1.02 | 0.99 – 1.05 |  |  |
| Social functioning (continuous)  TAAQOL subscale score | 92.1 | 75.6 | **0.98** | **0.96 – 0.99** | **0.97** | **0.96 – 0.99** |
| PSQI  Poor sleeper (no = ref) | 27.5 | 61.9 | **1.57** | **1.01 – 2.43** | **1.68** | **1.09 – 2.60** |
| Comorbidities  0  1-2  >2 | 49.6  44.8  5.7 | 34.5  51.5  13.9 | ref  1.24  1.22 | 0.80 – 1.93  0.55 – 2.70 |  |  |
| **Moderating factors** |  |  |  |  |  |  |
| Sex  Male  Female | 52.8  47.2 | 29.4  70.6 | ref  **2.71** | ref  **1.89 – 3.91** | ref  **2.45** | ref  **1.26 – 4.77** |
| Age at assessment (years)  18-29  30-39  ≥40 | 32.9  46.8  20.3 | 28.4  50.5  21.1 | ref  1.46  1.30 | ref  0.97 – 2.19  0.79 – 2.15 |  |  |
| Educational level  Low  Middle  High | 8.4  44.4  47.2 | 16.5  45.4  38.1 | ref  **0.50**  **0.41** | ref  **0.29 – 0.87**  **0.24 – 0.72** | ref  0.89  1.02 | ref  0.46 – 1.74  0.51 – 2.03 |
| Employment status  Employed  Not employed | 92.6  7.4 | 74.7  25.3 | ref  **3.78** | ref  **2.36 – 6.07** | ref  1.62 | ref  0.88 – 2.97 |
| Relationship status  In a relationship  Not in a relationship | 81.2  18.8 | 79.9  20.1 | 0.95  ref | 0.61 – 1.49  ref |  |  |

*Note: Insufficient statistical power make reliability of the results of the complete case analysis presented here questionable.*

*Abbreviations: CF=Chronic fatigue; CNS=Central Nervous System; BMI=Boddy Mass Index; HADS= Hospital Anxiety and Depression Scale; RSES=Rosenberg Self-Esteem Scale; CRP=C-Reactive Protein; TAAQOL= TNO (Netherlands Organisation for Applied Scientific Research) and AZL (Leiden University Medical Centre) Questionnaire for Adult’s Quality of Life; PSQI= Pittsburg Sleep Quality Inde;.95% CI= 95% Confidence Interval; n/a=could not be generated as too less cases were present for this particular variable.*

**Mean scores are shown for continues variables*

*^a^ Diagnostic groups included all malignancies covered by the third edition of the International Classification of Childhood Cancer (ICCC-3) as well as multifocal Langerhans cell histiocytosis.*

*^b^ Includes all morphology codes specified in the ICCC-3 under lymphomas and reticuloendothelial neoplasms, except for Hodgkin lymphomas. Also includes multifocal Langerhans cell histiocytosis.*

*^c^ Includes all morphology codes specified in the ICC-3 under other malignant epithelial neoplasms and malignant melanomas and other and unspecified malignant neoplasms.*

*^d^ Treatment data included primary treatment and all recurrences.*

*^e^ Three separate multivariable logistic regression models with Chronic fatigue as dependent variable and the assumed triggering, maintaining and moderating factors as independent variables. Each variable was adjusted for the other variables of the same group.*

*^f^ Chronic fatigue as dependent variable and the statistically significant (p<0.05) factors from the separate models ^e^ as independent variables in one “final model”. Each variable was adjusted for the other variables included in this final model.*
